# Association between Residence Location and Pre-Hospital Delay in Patients with Heart Failure

**DOI:** 10.3390/ijerph18126679

**Published:** 2021-06-21

**Authors:** Kyoung Suk Lee, Hyeongsuk Lee, Jae-Hyeong Park

**Affiliations:** 1 Center for Human-Caring Nurse Leaders for the Future by Brain Korea 21 (BK 21) Four Project, The Research Institute of Nursing Science, College of Nursing, Seoul National University, Seoul 03080, Korea; kyounglee@snu.ac.kr; 2 College of Nursing, Gachon University, Incheon 21936, Korea; 3 Department of Internal Medicine, Chungnam National University Hospital, School of Medicine, Chungnam National University, Daejeon 35015, Korea; jaehpark@cnuh.co.kr

**Keywords:** heart failure, rural population, care seeking, symptom experience

## Abstract

Rural residents with heart failure (HF) face more challenges than their urban counterparts in taking action when their symptoms worsen due to limited healthcare resources in rural areas. This may contribute to rural residents’ pre-hospital delay in seeking medical care. However, few studies have investigated the relationship between residence locations and pre-hospital delay among patients with HF. Therefore, this study determined whether living in rural areas is associated with pre-hospital delay in patients with HF. A retrospective electronic medical record review was conducted using the data of patients discharged with worsening HF from an academic medical center. Data on postal codes of the patients’ residences and their experiences before seeking medical care were obtained. Pre-hospital delay was calculated from the onset of HF symptoms to hospital arrival. A multivariate linear regression analysis was performed to determine the relationship between residence location and pre-hospital delay. The median pre-hospital delay time of all patients was 72 h (N = 253). About half of the patients did nothing to relieve their symptoms before seeking medical care. Living in urban areas was associated with a shorter pre-hospital delay. Patients with HF waited several days after first experiencing worsening of symptoms before getting admitted to a hospital, which may be related to inappropriate interpretation and responses to the worsening of symptoms. Furthermore, we found that rural residents were more vulnerable to pre-hospital delay than their urban counterparts.

## 1. Introduction

Recurrent hospitalizations are common in patients with heart failure (HF), and this increases direct and indirect costs of healthcare, leading to poor quality of life for patients with HF [1,2]. In the United States, the rate of 30-day rehospitalization was 20%, with 50% of readmissions being attributable to HF occurring within 13 days after discharge [3]. Pre-hospital delay is an essential factor that contributes to rehospitalization. Reportedly, the median time interval between recognizing the worsening of HF symptoms and seeking medical care ranges from 5.3 h to 7 days [4,5,6,7,8].

Inequitable rural access to health-relevant resources (e.g., distance to the nearest hospital or ratios of healthcare providers to population) results in poorer health status for people living in rural areas [9]. In the HF population, it has been reported that rural patients with HF are more likely to have a poor prognosis (e.g., rehospitalization) compared to their urban counterparts [10,11,12]. Since rural residents have limited access to healthcare resources such as medical care (e.g., because of long distance and lack of transportation), they are likely to face more challenges in taking actions than encountered by urban residents with HF when their symptoms worsen, which might lead to pre-hospital delay in seeking medical care. However, few studies have investigated the relationship between residence locations and pre-hospital delay among patients with HF. Therefore, it is essential to explore the association between residence location and pre-hospital delay in seeking medical care. In this study, we describe the experience of rural and urban patients with HF before seeking medical care and explore whether living in rural areas is associated with a pre-hospital delay in seeking medical care by the patients.

## 2. Materials and Methods

### 2.1. Design and Sample

A retrospective electronic medical record review was conducted using the data of patients discharged with a diagnosis of HF (International Classification of Disease-10 codes: 150.1–150.4 and 150.9) from an academic medical center in Korea between January 2014 and March 2017. Initially, 818 records were screened. About 512 records were excluded because the primary reason for the index hospitalization was not related to HF. Of the 306 records retained, 53 were further excluded since they did not include data about the patient’s symptoms on admission or indicate their discharge status (e.g., discharged to long-term care facilities). Thus, a total of 253 records were included in this study. An exemption from the institutional review board at the study site was obtained before conducting this study.

### 2.2. Procedure

Electronic medical records were reviewed to abstract the following information about pre-hospital experiences: postal codes of the patients’ residence, symptoms and signs experienced by patients before the index hospitalization, responses to worsening of symptoms and signs, and precipitating factors reported by patients. A data abstract protocol was developed to abstract data consistently. Any ambiguity was discussed with an expert in patient care.

### 2.3. Measurement

Residences were categorized into rural or urban areas based on the postal codes of the patients’ addresses [13]. Rural and urban areas were defined as the regions outside and within the boundary of the city where the study hospital was located.

A previous literature review of HF symptoms was used to generate a list of 17 symptoms and signs [14].

Possible actions taken by patients to relieve their symptoms and signs and the precipitating factors of HF exacerbation reported by patients were identified from previous studies [8,15]. Multiple answers were possible on the items regarding the actions taken by patients and the reported precipitating factors.

The demographic and clinical characteristics (e.g., age, left ventricular ejection fraction (LVEF), and New York Heart Association (NYHA) functional class), admission route, and vital signs on admission were also reviewed. Comorbidity was assessed using the Charlson comorbidity index (CCI), which is derived by summing the weights assigned to each comorbid condition [16]. Body mass index (BMI) was calculated by dividing the weight in kilograms on admission by the square of the height in meters.

The duration of pre-hospital delay (i.e., pre-hospital delay time) was defined as the time interval between a patient’s initial awareness of any symptoms or signs of HF and the patient’s hospitalization via the emergency department or clinic.

### 2.4. Data Analysis

To compare the patients’ characteristics, pre-hospital experience, and duration of pre-hospital delay in seeking medical care by rural/urban status, an independent *t*-test or chi-square test was conducted, as appropriate. Multivariate linear regression analysis was performed to determine whether rural/urban status predicted the duration of pre-hospital delay in seeking medical care. Since the distribution of the pre-hospital delay time was skewed, log-transformed values were used in the regression model. From the previous studies, the following covariates were included in the regression model: age, gender, marital status, education level, BMI, LVEF, NYHA functional class, CCI, admission route, HF-related admission before the index hospitalization, actions to relieve symptoms before seeking medical care (doing nothing vs. doing anything), and symptoms (i.e., shortness of breath, shortness of breath with activity, shortness of breath when lying flat, lower extremity swelling, coughing, and chest pain). Of the 17 symptoms, 6 were selected because at least 10% of the patients reported these symptoms before hospitalization. Statistical analysis was performed using SPSS 25.0 (IBM, Chicago, IL, USA). The statistical significance was set at *p* < 0.05.

## 3. Results

### 3.1. Sample Characteristics

Of the 253 patients admitted because of HF exacerbation, the majority were women (53.8%), married (80.2%), and had below high school level education (71.5%; Table 1). About 36 patients (14.2%) had been hospitalized more than once because of HF exacerbation in the last three months before the index hospitalization. Patients had been diagnosed with HF for an average of 3 years. More than two-thirds of the patients (68.8%) were admitted via the emergency room. Admission vital signs were within normal ranges, except for respiration rates and saturation.

About two-thirds of the patients (66.4%) lived in urban areas. Compared to patients residing in urban areas, those in rural areas were more likely to be older, have lower BMI, and be in NYHA functional class III/IV on admission (Table 1). Rural residents were more likely to have lower heart rate and systolic blood pressure than those of urban residents. However, regardless of rural and urban status, heart rate and systolic blood pressure were within normal ranges.

### 3.2. Duration of Pre-Hospital Delay

**Figure 1:**
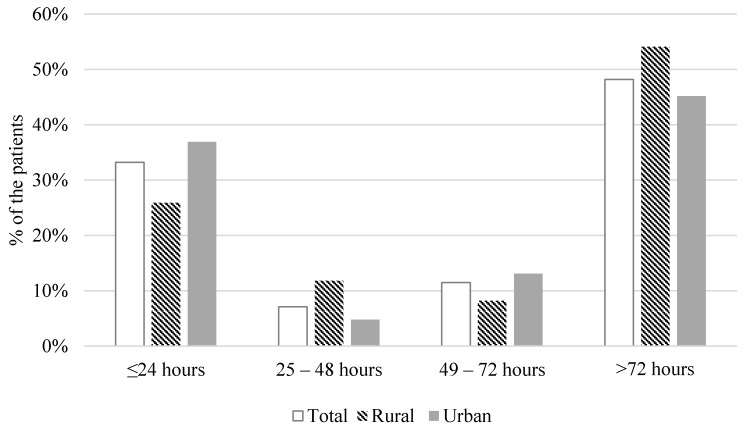
Distribution of the pre-hospital delay in seeking medical care (N = 253).

The median pre-hospital delay time from the onset of HF symptoms to hospital admission was 72 h (interquartile range: 17.25–240.00; Table 1). About one-third of the patients (33.2%) sought medical help within 24 h after HF symptoms developed. The pre-hospital delay time did not differ significantly between rural and urban patients with HF (mean of 410.2 h and 364.5 h, respectively). However, compared to urban patients, a larger proportion of rural patients were likely to seek medical care more than 72 h after and a smaller proportion were likely to seek medical care less than 24 h before the onset of HF symptoms (*p* = 0.045; Figure 1).

### 3.3. Symptom Experience before Seeking Medical Care

The average number of symptoms experienced before admission was 2.6 (SD: 1.6; Table 2). The most frequently reported symptom was shortness of breath (86.2%), followed by shortness of breath with activity (26.9%), lower extremity swelling (17.8%), coughing (16.6%), chest pain (15.8%), and shortness of breath when lying flat (14.6%). Almost none of the patients reported experiencing psychological symptoms before seeking medical care.

The number and types of symptoms experienced before the index hospitalization did not differ significantly by residential areas, except for lower extremity swelling. Rural patients were less likely than urban patients to have lower extremity swelling (*p* = 0.013).

### 3.4. Interpretation and Response to Worsening of Symptoms by Patients

Patients reported that their symptoms worsened due to comorbid conditions (26.5%) and nonadherence to medication (4.7%; Table 2). However, more than half of the patients were unable to identify the possible precipitating factors related to their symptoms (67.6%). Rural and urban patients with HF did not differ significantly in interpreting their worsening symptoms.

Patients consulted family members (27.3%) and/or their healthcare providers (9.5%) about their worsening symptoms (Table 2). About one-fourth of the patients visited physicians for issues unrelated to the heart (24.5%). To relieve their symptoms, one patient drank more water, while four patients consumed either nitrate sublingual or medicine for cold. However, about half of the patients did nothing before seeking medical care (50.6%), of which 14.1% waited until the next clinic appointment. The proportion of patients who did nothing and those who took some actions did not differ significantly between rural and urban residents (*p* = 0.79).

### 3.5. Factors Associated with a Pre-Hospital Delay

Living in urban areas (β = −0.126) predicted a shorter pre-hospital delay in seeking medical care after adjusting for covariates (Table 3). Among the covariates considered, admission via an emergency department (β = −0.251) and doing nothing to relieve symptoms before admission (β = −0.771) were associated with a shorter pre-hospital delay. Among the symptoms experienced before admission, the presence of lower extremity swelling and coughing was associated with a longer pre-hospital delay (β = 0.131 and 0.157, respectively), while the presence of chest pain was related to a shorter pre-hospital delay (β = −0.135).

## 4. Discussion

In this study, we examined the experiences of patients with HF before seeking medical care because of their HF exacerbation and explored the association between the residence location and pre-hospital delay in seeking medical care. We found that before seeking medical care, patients waited approximately 380 h (almost 16 days) after they first noticed worsening of symptoms, and about half of them did nothing to relieve their symptoms. Additionally, we found that living in rural areas was associated with a pre-hospital delay in seeking medical care. Our findings indicate that patients with HF experience difficulty in interpreting and responding to changes in their symptoms and signs of HF, which may result in pre-hospital delays in seeking medical care. Moreover, rural patients with HF were more vulnerable to pre-hospital delays in seeking medical care compared to urban residents with HF.

Patients with HF face substantial challenges in detecting and interpreting symptoms, which can result in taking ineffective actions to address their worsening symptoms [17]. Our study also demonstrated patients’ difficulty in interpreting their symptoms, which resulted in a significant pre-hospital delay in seeking medical care. Patients in our study experienced typical symptoms of HF (e.g., shortness of breath and lower extremity swelling). However, most of the patients were unable to identify their worsening symptoms, which subsequently led them either to take inadequate actions (e.g., visiting clinics other than cardiology clinics or taking unrelated medications) or do nothing. The proportion of patients who did not take any action (50.6%) was greater than that evidenced in previous studies, which ranged from 12.8 to 36.3% [6,8,18]. Although our sample patients did not take appropriate actions to relieve symptoms, the pre-hospital delay time before seeking medical care (a median of 72 h) was not significantly longer than that evidenced in previous studies conducted in the United States and Japan, which ranged from a median of 60 h to 124 h [5,6,7,8]. This may be related to the healthcare system in Korea where the entire population is covered by the national health insurance.

Patients with HF often experience various symptoms before admission, although the most frequently reported symptoms are dyspnea (55.0–93.0%), dyspnea with activity (42.0–92.9%), edema (38.5–64.0%), and fatigue (53.0–84.4%) [5,6,8,18]. This is consistent with our findings. However, except for shortness of breath, other symptoms and signs of HF, such as shortness of breath with activity and edema, were reported by a small number of patients (17.8% and 26.9% of the patients, respectively). When comparing the average number of symptoms experienced by patients in our study with those in the study by Sethares et al. (2014), in which the median pre-hospital delay time (of 60 h) was similar to ours, patients in our study reported a much smaller number of symptoms (2.6 vs. 12.2 symptoms) [7]. This may be because our patients were not aware of the symptoms and signs of HF; therefore, they did not monitor the symptoms and signs of HF daily and were unable to accurately report their symptom experiences to the clinicians who documented their medical history and records.

We found that rural patients with HF were more likely to have longer pre-hospital delay in seeking medical care than urban patients with HF, which can be explained by three reasons. First, patients’ lack of understanding of HF and its management can make it difficult for them to link their symptoms with HF. Although patients’ levels of understanding of HF and its relevant management could not be assessed through the electronic medical record review that we employed in this study, several results of our study suggest that rural residents had a limited understanding of HF. Approximately 82% of the rural residents in our study were unable to identify precipitating factors of worsening symptoms of HF or link their symptoms with HF, which consequently led them to do nothing or visit clinics other than cardiology clinics. The number of patients reporting edema was significantly lower in rural patients than in urban patients. This may indicate that rural residents did not understand that swelling is a sign of fluid overload and, in turn, were less likely to carefully check their swelling compared to urban residents. Furthermore, lower levels of education in our sample of rural residents compared to urban residents may have contributed to greater difficulty in comprehensively understanding HF and its management.

Second, the culture embedded in rural communities could contribute to pre-hospital delay in seeking medical treatment even if rural residents were aware of HF exacerbation. Since continuing work (e.g., working on land or garden) is valued in the rural setting, some rural residents believe that health is affected more by destiny than medical treatment and are reluctant to meet clinicians even when their symptoms worsen [19]. Third, an inaccessible rural healthcare system could contribute to pre-hospital delay even after rural dwellers with HF decide to seek medical care. The lack of public transportation and hospitals, longer distance to healthcare centers, and low density of physicians with higher reliance on generalists and high healthcare staff turnover in rural areas have been well documented in previous studies [20,21]. Although not addressed in this study, longer distances to the nearest hospital for rural residents could be a significant factor contributing to a longer pre-hospital delay in seeking medical care. Similar issues have also been reported in Korea, i.e., the number of medical facilities and healthcare providers is smaller in rural areas than in urban areas [22].

One interesting finding in our study was that prior admission experience because of HF was not a significant predictor of pre-hospital delay, although past experiences are an important facilitator in building expertise in self-care [17]. Unlike in the United States, discharge education for patients with HF is not a common practice in Korea. The discharge education for patients with HF in our study mainly comprised providing a list of medications and scheduling the next outpatient visit at the medical center where this study was conducted. Therefore, patients may not have gained enough opportunity to learn from their previous hospitalization experiences.

This study has some limitations. The data were obtained by utilizing electronic medical record reviews. Since the obtained information was dependent on clinicians’ documentation, certain information, such as precipitating factors (e.g., family income), might be incomplete. Given that this study was conducted in an academic medical center, our findings may not be generalizable to patients admitted to other hospitals. However, this academic medical center is located in a region with close proximity to both urban and rural areas, rendering it suitable to explore the association between the residence location and pre-hospital delay. Furthermore, the national health insurance covers the whole population living in Korea; therefore, our findings may not be applicable to nations with other medical insurance systems.

## 5. Conclusions

We observed that patients with HF waited several days after first experiencing worsening of HF symptoms before getting admitted to a hospital. The pre-hospital delay appears to be related to inappropriate interpretation and responses to worsening symptoms and signs of HF. Our results suggest that the limited understanding of HF and its management can be a possible reason for the pre-hospital delay and call for greater efforts to improve patients’ comprehensive understanding of HF and its management. Furthermore, we found that rural residents were more vulnerable to pre-hospital delays in seeking medical care compared to their urban counterparts. Further research should determine the factors that contribute to pre-hospital delays among rural patients with HF compared to their urban counterparts. This information is valuable for improving the outcomes in rural patients with HF.

## Figures and Tables

**Table 1:** Sample characteristics (N = 253).

Characteristic		Total	Rural Residents (*n* = 85)	Urban Residents (*n* = 168)	*p*-Value
		Mean (SD) or Frequency (%)			
Age (years)		71.5 (12.7)	74 (11.1)	70 (13.3)	0.018
Gender	Male	117 (46.2%)	39 (45.9%)	78 (46.4%)	0.934
	Female	136 (53.8%)	46 (54.1%)	90 (53.6%)	
Body mass index (kg/m^2^)		23.9 (4.1)	23.1 (4.0)	24.4 (4.1)	0.021
Education level	≥High school graduate	72 (28.5%)	16 (18.8%)	56 (33.3%)	0.016
Marital status	Single/widowed/divorced	50 (19.8%)	20 (23.5%)	30 (17.9%)	0.285
	Married	203 (80.2%)	65 (76.5%)	138 (82.1%)	
Charlson comorbidity index		2.8 (1.7)	2.6 (1.5)	3.0 (1.8)	0.086
Left ventricular ejection fraction	<40	129 (51.0%)	46 (54.1%)	83 (49.4%)	0.479
	≥40	124 (49.0%)	39 (45.9%)	8 (50.6%)	
Etiology of heart failure	Ischemic	90 (35.6%)			
	Non-ischemic	163 (64.4%)			
Years since HF diagnosis		2.8 (3.4)	3.3 (3.9)	2.5 (3.1)	0.072
NT-pro BNP (*n* = 214)		7266.5 (8625.7)	6361.3 (7435.2)	7744.9 (9182.1)	0.265
NYHA functional class on admission	II	55 (21.7%)	12 (14.1%)	43 (25.6%)	0.037
	III/IV	198 (78.3%)	73 (85.9%)	125 (74.4%)	
Prior admission due to HF exacerbation within 3 months		36 (14.2%)	11 (12.9%)	25 (14.9%)	0.677
Number of medications		6.9 (3.1)	6.6 (2.7)	7.2 (3.3)	0.117
ACEi/ARB		184 (72.7%)	62 (74.7%)	122 (74.4%)	0.958
Beta blockers		211 (83.4%)	70 (84.3%)	141 (86.0%)	0.730
Diuretics		225 (88.9%)	75 (90.4%)	150 (91.5%)	0.774
Admission route	Emergency room	174 (68.8%)	58 (68.2%)	116 (69.0%)	0.895
	Clinic	79 (31.2%)	27 (31.8%)	52 (31.0%)	0.895
Admission vital sign	Systolic blood pressure	138.6 (31.7)	132.8 (29.5)	141.6 (32.5)	0.038
	Diastolic blood pressure	81.0 (17.4)	80.1 (16.8)	81.4 (17.8)	0.585
	Heart rate	97.1 (24.2)	91.8 (21.7)	99.7 (25.0)	0.014
	Respiration	24.6 (6.0)	24.6 (5.9)	24.7 (6.0)	0.877
	Saturation	95.0 (5.4)	95.1 (6.2)	95.0 (5.0)	0.879
Time interval from onset to the admission (hours) ^1^		379.8 (1125.6)	410.2 (930.7)	364.5 (1214.7)	0.114
		Median: 72 (IQR: 17.25–240.00)	Median: 96 (IQR 24.0–324.0)	Median: 72 (IQR 11.25–240.0)	

^1^ The distribution of the pre-hospital delay in seeking medical care was skewed, and *p*-values were obtained based on the log-transformed values. Abbreviations: HF = heart failure; NT-pro BNP = N-terminal pro-brain natriuretic peptide; NYHA = New York Heart Association; ACEi/ARB = angiotensin-converting enzyme inhibitor/angiotensin II receptor blocker; IQR = interquartile range.

**Table 2:** Patients’ experiences before the index hospitalization (N = 253).

	Total	Rural Residents	Urban Residents	*p*-Value
	Mean (SD) or Frequency (%)			
Symptom experience				
Total number of symptoms experienced before the admission	2.6 (1.6)	2.6 (1.6)	2.7 (1.7)	0.807
Shortness of breath	218 (86.2%)	74 (87.1%)	144 (85.7%)	0.770
Shortness of breath with activity	68 (26.9%)	27 (31.8%)	41 (24.4%)	0.212
Lower extremity swelling	45 (17.8%)	8 (9.4%)	37 (22.0%)	0.013
Coughing	42 (16.6%)	9 (10.6%)	33 (19.6%)	0.068
Chest pain	40 (15.8%)	13 (15.3%)	27 (16.1%)	0.873
Shortness of breath when lying flat	37 (14.6%)	10 (11.8%)	27 (16.1%)	0.360
Poor appetite	19 (7.5%)	8 (9.4%)	11 (6.5%)	0.414
Difficulty in sleeping	16 (6.3%)	6 (7.1%)	10 (6.0%)	0.733
Palpitations	14 (5.5%)	5 (5.9%)	9 (5.4%)	0.863
Weight gain	13 (5.1%)	2 (2.4%)	11 (6.5%)	0.229
Dizziness	11 (4.3%)	6 (7.1%)	5 (3.0%)	0.133
Abdominal bloating	3 (1.2%)	0 (0.0%)	3 (1.8%)	0.553
Fatigue	3 (1.2%)	2 (2.4%)	1 (0.6%)	0.262
Nausea and vomiting	3 (1.2%)	0 (0.0%)	3 (1.2%)	0.049
Worrying	1 (0.4%)	1 (1.2%)	0 (0.0%)	0.336
Feeling sad or depressed	0 (0.0%)	0 (0.0%)	0 (0.0%)	N/A
Difficulty in concentrating or forgetfulness	0 (0.0%)	0 (0.0%)	0 (0.0%)	N/A
**Precipitating factors of symptoms identified by patients**				N/A
Unable to identify	171 (67.6%)	60 (70.6%)	111 (66.1%)	
Exacerbation of comorbid conditions	67 (26.5%)	18 (21.2%)	49 (29.2%)	
Non-adherence to medication	12 (4.7%)	5 (5.9%)	7 (4.2%)	
Psychological stress	2 (0.8%)	1 (1.2%)	1 (0.6%)	
Loss of clinic appointment	1 (0.4%)	1 (1.2%)	0 (0%)	
**Actions taken to decrease symptoms before the hospitalization ^1^**				N/A
Doing nothing	128 (50.6%)	42 (49.4%)	86 (51.2%)	
Went to clinic other than cardiology clinic	62 (24.5%)	25 (29.4%)	37 (22.0%)	
Call to family	69 (27.3%)	23 (27.1%)	46 (27.4%)	
Call to the doctor	24 (9.5%)	9 (10.6%)	15 (8.9%)	
Took unrelated medication or actions irrelevant to HF	5 (2.0%)	1 (1.2%)	4 (2.4%)	
Took an extra diuretic	1 (0.4%)	0	1 (0.6%)	

^1^ Multiple answers were given; HF = heart failure.

**Table 3:** Factors associated with pre-hospital delay in seeking medical care (N = 253).

Variables	Unstandardized Coefficients	Standardized Coefficients	*p*-Value	95% Confidence Interval
Urban residence (vs. rural residence)	−0.563	−0.126	0.034	−1.082, −0.043
Age (years)	−0.003	−0.016	0.806	−0.025, 0.019
Female (vs. male)	0.120	0.028	0.635	−0.377, 0.616
Body mass index (kg/m^2^)	0.061	0.118	0.061	−0.003, 0.124
≥High school graduation (vs. <high school education)	0.283	0.061	0.343	−0.304, 0.87
Married (vs. single/divorced/widowed)	−0.576	−0.109	0.059	−1.176, 0.023
Preserved ejection fraction (vs. reduced rejection fraction)	−0.292	−0.069	0.227	−0.766, 0.183
NYHA functional class III/IV (vs. II)	0.289	0.057	0.348	−0.317, 0.895
Charlson comorbidity index	−0.088	−0.072	0.232	−0.232, 0.056
Admission via clinic (vs. emergency department)	1.286	0.283	0.000	0.755, 1.816
Prior admission experience due to HF exacerbation	−0.326	−0.054	0.346	−1.007, 0.354
Did nothing to relieve symptoms before seeking care (vs. did something)	0.771	0.183	0.002	0.294, 1.248
Shortness of breath	0.388	0.064	0.283	−0.322, 1.099
Shortness of breath with activity	0.387	0.082	0.178	−0.178. 0.952
Lower extremity swelling	0.722	0.131	0.027	0.084, 1.359
Coughing	0.891	0.157	0.007	0.24, 1.541
Chest pain	−0.778	−0.135	0.023	−1.450, −0.107
Shortness of breath when lying flat	0.093	0.352	0.791	−0.600, 0.787

Adjusted R^2^ = 0.231; model *p*-value < 0.001. Abbreviations: HF = heart failure; NYHA = New York Heart Association.

## Data Availability

The data are not publicly available due to their containing information that could compromise the privacy of research participants.
